# The #SeePainMoreClearly Phase II Pain in Dementia Social Media Campaign: Implementation and Evaluation Study

**DOI:** 10.2196/53025

**Published:** 2024-02-08

**Authors:** Louise I R Castillo, Vivian Tran, Mary Brachaniec, Christine T Chambers, Kelly Chessie, Alec Couros, Andre LeRuyet, Charmayne LeRuyet, Lilian Thorpe, Jaime Williams, Sara Wheelwright, Thomas Hadjistavropoulos

**Affiliations:** 1 Department of Psychology and Centre on Aging and Health University of Regina Regina, SK Canada; 2 Centre on Aging and Health University of Regina Regina, SK Canada; 3 Department of Psychology and Neuroscience Dalhousie University Halifax, NS Canada; 4 Centre for Pediatric Pain Research IWK Health Halifax, NS Canada; 5 Department of Pediatrics Dalhousie University Halifax, NS Canada; 6 Santa Maria Senior Citizens Home Regina, SK Canada; 7 Faculty of Education University of Regina Regina, SK Canada; 8 Department of Community and Epidemiology University of Saskatchewan Saskatoon, SK Canada; 9 Trusted Marketing Services Saskatoon, SK Canada

**Keywords:** knowledge translation, Twitter, older adults, Facebook, knowledge mobilization

## Abstract

**Background:**

Social media platforms have been effective in raising awareness of the underassessment and undertreatment of pain in dementia.

**Objective:**

After a successful pilot campaign, we aimed to scale our pain-in-dementia knowledge mobilization pilot initiative (ie, #SeePainMoreClearly) to several social media platforms with the aid of a digital media partner. The goal of the initiative was to increase awareness of the challenges in the assessment and management of pain among people with dementia. A variety of metrics were implemented to evaluate the effort. Through this work, we endeavored to highlight key differences between our pilot initiative (which was a grassroots initiative), focusing largely on Twitter and YouTube, and the current science-media partnership. We also aimed to generate recommendations suitable for other social media campaigns related to health or aging.

**Methods:**

Evidence-based information about pain in dementia was summarized into engaging content (eg, videos) tailored to the needs of various knowledge users (eg, health professionals, families, and policy makers). We disseminated information using Facebook (Meta Platforms), Twitter (X Corp), YouTube (Alphabet Inc), Instagram (Meta Platforms), and LinkedIn (LinkedIn Corp) and measured the success of the initiative over a 12-month period (2020 to 2021). The evaluation methods focused on web analytics and questionnaires related to social media content. Knowledge users’ web responses about the initiative and semistructured interviews were analyzed using thematic analysis.

**Results:**

During the course of the campaign, >700 posts were shared across all platforms. Web analytics showed that we drew >60,000 users from 82 countries to our resource website. Of the social media platforms used, Facebook was the most effective in reaching knowledge users (ie, over 1,300,000 users). Questionnaire responses from users were favorable; interview responses indicated that the information shared throughout the initiative increased awareness of the problem of pain in dementia and influenced respondent behavior.

**Conclusions:**

In this investigation, we demonstrated success in directing knowledge users to a resource website with practical information that health professionals could use in patient care along with pain assessment and management information for caregivers and people living with dementia. The evaluation metrics suggested no considerable differences between our pilot campaign and broader initiative when accounting for the length of time of each initiative. The limitations of large-scale health campaigns were noted, and recommendations were outlined for other researchers aiming to leverage social media as a knowledge mobilization tool.

## Introduction

### Background

Social media platforms play an important role in academic dissemination and have untapped potential as knowledge mobilization (KM) tools that can allow researchers to interact directly with the public worldwide [1]. KM encompasses activities involved in the synthesis and dissemination of research information; this process includes knowledge synthesis, dissemination, transfer, exchange, and cocreation by researchers and knowledge users [2]. Knowledge users, such as older adults and their families, are increasingly turning to web-based platforms (eg, Twitter [subsequently rebranded as X] and Facebook) to share and obtain information [3,4]. Health-related initiatives on social media networks have successfully raised awareness about a host of topics (eg, mental health, breastfeeding, and cancer) [5]. For example, a nationwide HIV prevention campaign (#PrEP4Love) garnered >40 million views across various social media platforms [6].

Researchers have supplemented social media KM initiatives with other web-based KM methods (eg, web-based repositories, educational videos, and community groups) [7,8]. Despite the innovation of this approach, the sustainability of social media health campaigns is contingent upon different factors (eg, continued content generation and ongoing funding) [9]. As such, there is a growing need for partnerships with digital media experts to aid in health KM. A notable example of this is the work by Chambers [10], who mobilized knowledge about pediatric pain within the context of a science-media partnership through her #ItDoesntHavetoHurt campaign. The initiative demonstrated worldwide impact and great success in producing and disseminating digital evidence–based content tailored to the needs of parents and health professionals, obtaining 1.3 million Twitter impressions, 5.5 million hashtag mentions, and >250,000 views of her YouTube video [10]. Impact was also demonstrated through indices of adoption and changed practices [10].

Although researchers have attempted to track the reach and impact of social media for KM through web metrics and the use of social listening software [11,12], very little research has been conducted using social media for KM in older adults with dementia. To address limitations in the reach of traditional KM campaigns and a gap in the literature, we launched and evaluated the pilot #SeePainMoreClearly (phase I) social media KM campaign with messages that reached >2,376,853 unique individuals on Twitter [9]. The goal of the pilot initiative was to increase awareness of the challenges in the assessment and management of pain among people with moderate to severe dementia with limited ability to communicate [13,14]. Moreover, we aimed to disseminate information on evidence-based approaches to effectively assess and manage pain in this population (eg, [15-18]). To maximize uptake, we created a web-based repository of pain assessment and management information [19]. We also prepared an engaging 2-minute YouTube video with evidence-based information about pain in dementia [20].

The script for the video was created and modified with input from health professionals, caregiver partners, researchers, and knowledge user organizations. The pilot campaign was evaluated by tracking social media and web metrics and by conducting qualitative analyses of social media posts in response to the #SeePainMoreClearly campaign over a 5-month period. #SeePainMoreClearly demonstrated substantial reach with >5,000,000 hashtag impressions on Twitter. The short video was viewed >50,000 times, and our web-based repository was visited by people in >55 countries. Moreover, the content analysis of social media posts (ie, tweets) from users who used the hashtag or responded to our messaging and content posted on Twitter were favorable. Many users expressed support for the initiative and increased advocacy for improved pain care for people living with dementia.

Our findings showed the effectiveness of web-based KM methods in reaching very broad international audiences quickly. Perhaps most importantly, without counting the posts that were produced by members of our team, the initiative doubled the number of posts made on the topic of pain dementia on Twitter during the campaign period as compared with a control period of the previous year [9]. It is important to note that the #SeePainMoreClearly pilot campaign took place in 2019, before the COVID-19 pandemic. The pilot #SeePainMoreClearly was a grassroots initiative in which a small number of researchers prepared engaging materials and disseminated them on their own with support from several knowledge user organizations that agreed to help disseminate the message. A lesson from the pilot #SeePainMoreClearly experience was that although KM grassroots campaigns can be very successful, lengthier social media campaigns (run solely by researchers) would not be sustainable, as involvement can be very time-consuming for researchers who generally must attend to multiple other obligations. A partner with digital media expertise and time to dedicate resources is necessary for a sustainable large-scale KM campaign. Hence, we sought and obtained funding to support such a partnership.

### Objectives

The primary purpose of this study was to launch and evaluate a longer (12 months instead of 5 months) #SeePainMoreClearly campaign with expanded social media platform coverage (eg, Twitter, Instagram, Facebook, and LinkedIn as opposed to just Twitter) with the following additional objectives:

1. Track the reach of a larger social media KM campaign over a 12-month period on various platforms (eg, Facebook, Twitter, Instagram, and LinkedIn)
2. Evaluate the impact of the campaign on knowledge users’ (eg, patient, caregiver, health professional, and policy maker) knowledge
3. Evaluate the impact of a science-media partnership on our pilot campaign in meeting knowledge users’ needs
4. Outline recommendations to develop and scale social media KM initiatives.

We expected the results to demonstrate a large scope and breadth of our campaign, resulting in a large number of visits to our resource website [19]. Moreover, we predicted that our results would demonstrate the value of a science-media partnership as a strategy for improving KM in the area of pain in dementia. We contrast the experiences of the digital media partnership campaign with the pilot #SeePainMoreClearly campaign. The findings from this project can inform subsequent evaluations of social media as a KM tool.

## Methods

### Ethical Considerations

The evaluation process involving contact with human participants (eg, questionnaires and semistructured interviews) was approved by the University of Regina Research Ethics Board (#REB 2020-036).

### Identification and Engagement of Digital Media Partner

Given the nature of our funding, we were restricted to identify a digital media partner within our home province. As such, a web search was conducted to identify potential locally based digital media partners that listed social media promotion as part of their services. We then communicated with 2 of the firms that, based on their websites, seemed highly experienced with social media marketing and described the nature of our work to confirm that they understood the needs of our project and that they were interested in partnering with us for the campaign. Our institution required us to subject the project to an open competitive bidding process before awarding the contract. This process allows qualified bidders to submit proposals and budgets. The 2 firms with which we had communicated were invited to submit their proposals. On the basis of this process, the best qualified bidder who offered competitive pricing was selected from a total of 3 bidders.

### Development of Content and the Campaign

**Figure 1:**
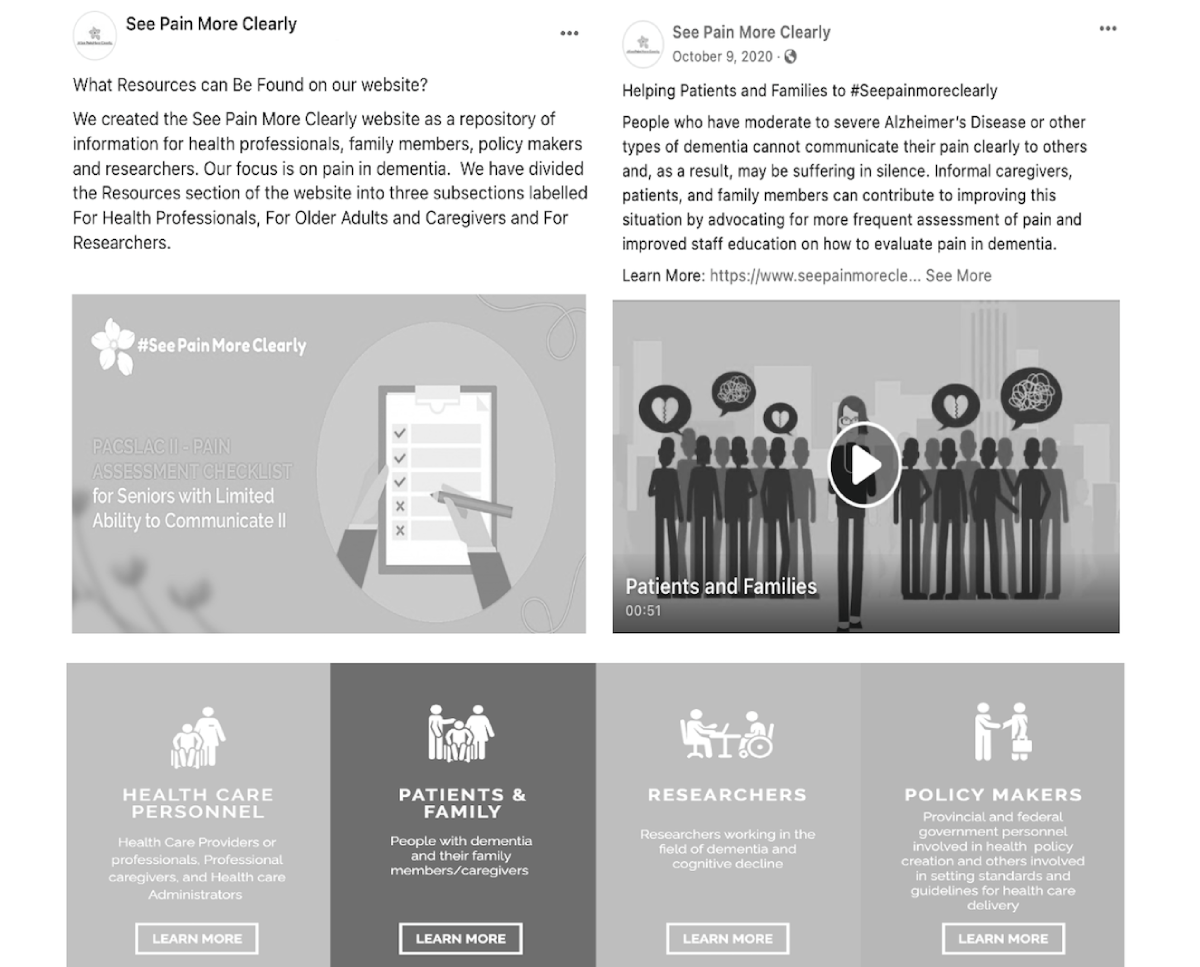
Digital content samples shared throughout the initiative.

We worked with the selected digital media partner to develop the campaign content and KM strategy as well as to collect social media analytics in response to our campaign. An iterative process was implemented with team collaborators (eg, caregiver partners, researchers with knowledge of the content area, media experts, and health care professionals) with the aim of developing lay summaries of evidence-based information about pain in dementia. Specifically, a series of web-based meetings with members of the team were coordinated to identify pertinent information about pain assessment and management among older adults with dementia, which served as the basis of the campaign’s content. Accordingly, the general topics agreed upon by the team were as follows: (1) the feasibility of regular pain assessments, (2) available guidelines for pain assessment, (3) resources for informal caregivers, (4) effects of psychotropic medication in long-term care (LTC), (5) validated pain assessment tools, (6) implementing regular pain care in LTC, (7) gaps in education for health professionals and ways to address this challenge, (8) effects of untreated pain in dementia, (9) pain care during the COVID-19 pandemic, (10) cost of untreated pain for the health care system, (11) benefits of regular pain assessments, and (12) effective ways to assess pain in dementia. Once the general topics were identified, information was gathered that corresponded to established guidelines (eg, [17,18,21,22]) or was supported by published research in leading peer-reviewed journals. Specific evidence-based information was selected by our team, which included content experts and caregivers with lived experience. Next, the team developed lay summaries (eg, 200-300 words) of the evidence present in the literature covering each aforementioned topic (eg, [17,18,21,22]). Several rounds of refinement were conducted by team members. Following this, the team developed 8 cross-cutting messages and key points (Multimedia Appendix 1) divided by the target group (eg, health professionals, policy makers, and public or families). These messages were then relayed to the digital media team as the basis for the messaging developed for the campaign. The digital marketing team developed a series of images and posts for each target group. Moreover, a short animated (ie, 2-minute long) video was created for each target group (eg, family, health care professionals, researchers, and policy makers). The animated videos were posted on the web page and on social media platforms. Examples of the content and posts are shown in Figure 1. Blog posts written by team members and people with lived experiences related to the topics outlined in this section were shared throughout the initiative. A total of 42 blog posts [23] were created and posted on the web page of this project.

### Dissemination Strategy

Unlike the pilot campaign [9], which focused on Twitter and YouTube, information was disseminated on 5 social media platforms used for the campaign: Facebook (Meta Platforms), Twitter (X Corp), YouTube (Alphabet Inc), Instagram (Meta Platforms), and LinkedIn (LinkedIn Corp).

A total of 756 posts were shared across Facebook, Twitter, Instagram, and LinkedIn, and 5 videos were posted on YouTube over a 12-month period (ie, October 1, 2020, to September 30, 2021). A key goal of the dissemination strategy was to direct knowledge users to the web-based repository to share further information and resources about pain in dementia [19]. We designed the content (eg, social media posts, images, and short animated videos) to be of interest to our target groups. Moreover, we developed social media posts to gain the attention of specific target groups (eg, by including an image with the statement *Resources for researchers* prominently displayed). As with the pilot initiative, the #SeePainMoreClearly hashtag was used to maximize the reach of the content and information. We leveraged events (eg, the International Day of Older Persons in October, World Alzheimer Awareness Month in September, and Alzheimer Awareness Month in January) to develop tailored content. The total amount of money spent on post promotion and advertisements was CAD $29,793.86 (US $22,133.06); the vast majority of this amount was spent on Facebook and Instagram, CAD $26,384 (US $19,599.96), Twitter, CAD $3045 (US $2262.05), and LinkedIn, CAD $364.86 (US $271.04).

### Evaluation of the Campaign

#### Engagement and Reach of the Initiative

##### Web Analytics

A longitudinal data collection strategy was used to obtain web and social media metrics over a 12-month campaign period (ie, October 1, 2020, to September 30, 2021). For the website, the number of content viewers and pages views were tracked using Google Analytics [24]. For our analysis, we reported a descriptive count (eg, count of total engagement, reach, and impressions) of all web and social media analytics. Google Analytics was used to collect data from the website. To clarify how Google Analytics works, each time a user visits a web page, a tracking code by Google Analytics collects information on how that user interacted with the page [25]. Google Analytics then aggregates and organizes this information in the Google Analytics portal as a report detailing different metrics (eg, the count of web page visits and the number of pages viewed). Similarly, the social media metrics were aggregated by each social media platform. This information was accessed through the social media platform’s website (eg, Facebook Analytics). Web and social media analytics were exported and reported.

##### Social Media Analytics

The reach, scope, and engagement of the initiative and web-based repository were monitored through social media metrics (eg, web analytics, hashtag analytics, and social media analytics). Similar metrics have been used in previous research to quantify the impact of social media initiatives [10,12]. The number of impressions (ie, the number of times users have seen the web-based content), reach (ie, the number of unique users who have seen the web-based content), and engagement (ie, the number of comments, retweets, “likes,” and shares) were extracted using the analytics provided by each platform. Not all analytics were available for each social media platform (eg, reach was not available for LinkedIn). Analytics were summarized based on three sources of information: (1) analytics of only paid posts provided by the social media platform, (2) analytics of the total number of posts (ie, paid and unpaid) provided by the social media platform, and (3) third-party social media analytics manager (eg, CloudCampaign) provided by the digital marketing team. Moreover, our digital media partner could only obtain data for specific periods (eg, year-long data could not always be obtained) for some platforms owing to limitations in the sources of information. Nevertheless, attempts were made to encompass the entire evaluation period. Depending on the data source, data were provided from (1) October 1, 2020, to September 3, 2021; (2) October 1, 2020, to November 2, 2020; or (3) November 3, 2020, to September 30, 2021. Thus, the reported numbers varied as a function of the period. The Keyhole social media monitoring (also known as social listening) software [26] was used to collect the metrics and analytics for the #SeePainMoreClearly hashtag on Twitter.

#### Knowledge Users’ Responses to the Initiative

##### Semistructured Interviews With Knowledge Users

Individuals who viewed and engaged with the content (eg, responded to social media posts or added their own commentary when reposting content shared throughout the initiative) on social media were invited to participate (ie, research personnel commented back to users who responded to social media posts and invited them to participate in interview) in semistructured interviews with a researcher to gain an understanding of their perceptions about the initiative. Moreover, invitations (ie, through social media posts or recruitment posters) to participate in the interview were circulated across social media platforms. All participants recruited through social media posts were asked to contact the research team to indicate their interest. The interviews were conducted over Zoom (Zoom Video Communications Inc) audio-only and covered the following topics: perceptions about the initiative, quality of information and messages disseminated, and impact of the initiative on knowledge and behavior (Multimedia Appendix 2). Zoom’s automated transcription feature was used to facilitate the transcription of the interviews. The transcription was then reviewed with the interview audio recording to ensure accuracy. NVivo (version 12; Lumivero) software [27] was used to facilitate the coding reliability thematic analysis [28]. Two independent researchers initially organized a subset of data into clusters based on commonalities and recurring ideas to develop a coding book [28]. Consistent with a coding reliability thematic analysis approach, themes were developed inductively and aligned with the data collection questions of the study [28]. Themes that emerge from this analysis can be viewed as a summary of the participant responses. Codes were developed after data familiarization, while keeping the data collection questions in mind. Two coders individually coded a subset (3/13, 20%) of the data to develop a coding book. They grouped each line of text into clusters and then into higher-level codes (ie, themes). The coders then met to discuss and finalize the coding book. Discrepancies between the coding researchers and decisions to merge or split categories were discussed throughout the process until a consensus was reached. The developed coding book was applied to the remaining data to identify the prevalent categories and obtain a frequency count of the identified themes. To ensure rigor in our analysis, we conducted consistency checks on the coded themes and categories (eg, discussing discrepancies between coders) and maintained ongoing communication among coders throughout the process to establish consensus. Moreover, a randomly selected subset of data was coded to assess intercoder agreement. Triangulation was considered through the different perspectives (eg, health professionals, caregivers, and people with dementia) obtained in this study and the use of a mixed methods approach (ie, use of interview and numeric questionnaire data). A primary coder coded the entire data set, and a secondary coder coded a subset of the data to establish intercoder agreement. The second coder coded a randomly selected 20% (3/13) of the participant data. NVivo calculates a κ coefficient to evaluate agreement as follows: total units of agreement between the 2 coders minus the expected frequency of the agreement occurring by chance, divided by the total units within the source minus the expected frequency of the agreement occurring by chance [29].

##### Social Media Comments About the Campaign

Social media analytics do not provide information about the content of the comments in response to our initiative (ie, whether users liked or disliked the content). As such, we analyzed the responses to the initiative to better understand users’ perception of our campaign. To assess the perception of a wider sample of knowledge users, comments by web users in response to the posts made during the campaign across Facebook, Twitter, Instagram, and LinkedIn were collected manually and subjected to a coding reliability thematic analysis, consistent with the method outlined in the *Semistructured Interviews With Knowledge Users* section. The aim was to understand the type of comment (ie, opinion, personal experiences, and information) in response to the content shared to determine prevalent themes.

##### Overview of Evaluation Questionnaires

A brief survey was circulated across the campaign’s social media platforms to receive feedback about users’ perception of the content and information shared during the initiative. The participants were recruited via invitations on social media platforms. For example, social media posts were created to encourage participants to provide their input on the initiative by clicking on the link that directed users to the questionnaires. The link to the questionnaire was also available on the website for this project. Individuals who accessed the surveys were asked to indicate the group they belonged to (1) caregiver, family member, or friend of a person with dementia, (2) person living with dementia, (3) public, (4) health professional, or (5) policy maker. Demographic information questions about participant age, gender, country, relationship with the person living with dementia, and type of health care provider (ie, for individuals who identified as a health care professional) were collected and outlined using descriptive and frequency statistics.

##### Pain in Dementia Evaluation Questionnaire

A short questionnaire, used in the pilot campaign evaluation [9], was used to solicit anonymous responses from various knowledge users. The survey included both general and specific questions for the general public, health professionals, and caregivers and family of people with dementia. Most responses were provided along 7-point Likert scales (eg, 1=not very likely to 7=very likely) and focused on viewers’ opinions about the initiative, content, and likelihood of using the information provided. Descriptive statistics were calculated for the Likert scale items in the evaluation questionnaire.

##### Information Assessment Method for All

Users’ perception of the content and potential benefit of the information were assessed using the IAM4all (Information Assessment Method for All) [30]. The IAM4all consists of 6 general questions assessing information relevance and use, with nested follow-up questions depending on participant responses. Accordingly, the IAM4all is a 28-item self-report questionnaire designed to measure 4 levels of outcomes associated with receiving or seeking web-based information: situational relevance, cognitive impact, information use, and health benefits. Each item is rated based on agreement to a question (ie, yes, no, or possibly). Content validity of the IAM4all has been substantiated through a review of the theories of information science and feedback from knowledge users (eg, laypersons, health professionals, and experts) [30]. Descriptive and frequency statistics were used to calculate the means and SDs for the items in the questionnaire.

##### Web-Based Discussions About Pain in Dementia

The level of web-based discussions about pain in dementia was determined by examining the number of posts on Twitter and Facebook. Three periods of comparison were established: (1) a precampaign period (ie, a 7-month period following the end of the pilot campaign and before the start of #SeePainMoreClearly phase II, March 1, 2020, to September 30, 2020); (2) a campaign period (October 1, 2020, to September 30, 2021); and (3) a postcampaign period (ie, a corresponding 7-month period following the end of #SeePainMoreClearly phase II, October 1, 2021, to April 30, 2022). Searches for unique (ie, not retweets or reposts) tweets on Twitter containing the hashtags “#pain #dementia” AND/OR key words “pain dementia” were conducted for all 3 periods using the Keyhole software. A catalog of all the tweets during the specified periods was collated on a spreadsheet by Keyhole. A similar method could not be used to obtain Facebook posts on Keyhole (ie, this feature was not available on Keyhole for Facebook posts); hence, a manual search using the aforementioned keywords was conducted for all 3 periods. That is, the specific keywords were typed in the search bar on Facebook, and the period (eg, precampaign period, campaign period, and postcampaign period) was specified in the search filter. Posts for each platform across the 3 periods were cataloged. The following types of posts were excluded: (1) pertaining to pain as a metaphor (ie, using “pain” as a metaphor for psychological distress, such as in the context of grief “I feel your pain”), (2) unrelated to the problem of pain in dementia, (3) shared by our own research group, and (4) not in the English language.

## Results

### Engagement and Reach of the Initiative

#### Web Analytics

Over 60,000 users from 82 countries viewed the web-based repository website, which resulted in 83,247 page views over the 12-month period (ie, October 1, 2020, to September 30, 2021). In examining the manner (ie, how people found the website) in which users were directed to the website, a majority of users (ie, 51,693 users) were directed to the website through links (ie, posts including a link to the website) from social media platforms. Other users directly typed the website link (ie, 6927 users) or searched the website link (ie, 2152). A small number of website users (ie, 116 users) were referred by other sites (ie, partnering organizations linking the web-based repository website on their web page). The blog web pages were viewed 59,919 times. Of the blogs posted during the project, the article written (on the request of our team) by an acclaimed author, Louise Penny, in which she relayed her experiences caring for her husband who lived with dementia [31], garnered most of the views (ie, received 33,226 views).

#### Social Media Analytics

The analytics are summarized in Table 1 based on the 3 sources of information and data periods described in the *Methods* section. The advertisements that were initiated on Facebook could also be posted on Instagram based on advertisement specifications. To maximize reach, the digital media team specified advertisements on Facebook and Instagram to be displayed on all available placements and to the specified targeted audiences. This includes Facebook and Instagram feeds (ie, advertisements displayed on the user’s feed), in-stream advertisements for videos, search results, and external apps and sites (ie, advertisements displayed to audiences on additional websites and mobile apps, such as newspaper websites). That is, advertisements initiated on Facebook could be displayed on Facebook, Instagram, or to external websites or apps connected to Facebook, resulting in increased impression and reach than organically derived engagement. The advertisements analytics (eg, impression and reach) on Facebook and Instagram differs from Twitter and LinkedIn as a function of the data source (ie, paid impression or reach on Facebook and Instagram are higher because they capture not just impression or reach derived on Facebook and Instagram but also on third-party websites such as newspaper websites; Table 1).

**Table 1:** Summary of the social media analytics for the #SeePainMoreClearly campaign.

Period, metric, and source of information^a^				Facebook, n		Twitter, n		Instagram, n		LinkedIn, n
**Period 1^b^**										
	**Impressions**									
		Facebook overall analytics^c^	4,100,000		—^d^		—		—	
		Twitter only analytics^e^	—		724,200		—		—	
		Instagram only analytics^e^	—		—		4883^f^		—	
		LinkedIn advertisement analytics^g^	—		—		—		8538	
		Twitter advertisement analytics^g^	—		365,935		—		—	
	**Reach**									
		Instagram advertisement analytics^h^	—		—		138,833		—	
		Instagram only analytics^e^	—		—		4420		—	
		Facebook only analytics^i^	1,313,485		—		—		—	
	**Engagement**									
		Facebook only analytics^i^	282,704		—		—		—	
		Cloud Campaign^j^	—		—		977		—	
**Period 2^k^**										
	**Impressions**									
		Facebook only analytics^i^	519,929		—		—		—	
	**Reach**			—		—		—		—
	**Engagement**									
		Twitter only analytics^e^	—		630		—		—	
**Period 3** ^l^										
	**Impressions**									
		Cloud Campaign^i^	871,800		—		—		1267	
	**Reach**			—		—		—		—
	**Engagement**									
		Cloud Campaign^j^	—		2125		—		193	

^a^Some analytics could not be obtained for specific platforms because they were not available. Moreover, the digital marketing team could only obtain data for specific periods (eg, year-long data could not be obtained) for some platforms owing to limitations in the sources of information. Nonetheless, efforts were made to capture the entire period.

^b^Period 1=October 1, 2020, to September 30, 2021.

^c^“Facebook overall analytics”=generated from paid and unpaid impressions on Facebook and third-party websites such as newspaper websites that are part of the Facebook advertising display network across Canada.

^d^Data for this period or metric were not applicable or not available.

^e^“Twitter/Instagram/LinkedIn only analytics”=analytics based on unpaid posts circulated only on Twitter, Instagram, or LinkedIn.

^f^On Instagram, the period 1 impressions only include impressions for unpaid posts posted on Instagram. It does not include paid posts that were posted on Facebook but reached Instagram audiences.

^g^“Twitter/LinkedIn advertisement analytics”=analytics based on paid posts circulated on Twitter and LinkedIn.

^h^“Instagram advertisement analytics”=the analytics are based on paid posts that were posted on Facebook but reached Instagram audiences based on specified target audiences.

^i^“Facebook only analytics”=based on paid and unpaid posts that were circulated only on Facebook.

^j^CloudCampaign (ie, third-party social media monitoring software): these analytics are based on unpaid posts circulated individually on Twitter, LinkedIn, and Instagram. The Cloud Campaign analytics from Facebook includes analytics of paid and unpaid posts circulated only within Facebook.

^k^Period 2=October 1,2020, to November 2, 2020.

^l^Period 3=November 3,2020, to September 30, 2021.

For a graphical illustration of the analytics, refer to Multimedia Appendix 3. Social media analytics indicated that 1,313,485 people were reached by the content shared on the Facebook page created for this project (Table 1). The #SeePainMoreClearly hashtag on Twitter (eg, obtained by Keyhole) was used by 540 users, resulting in 2835 posts. Moreover, the hashtag reached 1,691,440 users and gained 8,592,929 impressions and 8696 engagements. The 4 animated videos developed during the initiative were viewed over 257,000 times across the web page and social media platforms. The views for each video were as follows: 106,055 (family), 102,407 (health care professionals), 42,731 (policy makers), and 5911 (researchers). The pilot campaign video was also disseminated during phase II of the campaign across social media platforms and received 45,637 views (ie, 43,870 views on Facebook, 1043 on Twitter, 709 views on YouTube, and 15 views on Instagram).

### Knowledge Users’ Responses to the Initiative

#### Semistructured Interviews

##### Overview

A total of 13 knowledge users who interacted with the initiative’s posts participated in semistructured interviews. All participants provided informed consent. Participants had varied perspectives: 1 participant had lived experience of dementia, 4 participants had family members living with dementia and were health professionals, 4 participants were caregivers, and 4 were clinicians. A thematic analysis was conducted by 2 separate coders to establish an intercoder agreement. A randomly selected 20% (3/13) subset was coded by a second coder to assess for agreement. Overall agreement was excellent (*k*=0.81). Five themes emerged from the data: (1) increased awareness about the problem of pain in dementia, (2) perceived barriers to pain management, (3) increased knowledge and changes in behavior, (4) value of social media as a method of scientific dissemination, and (5) suggestions for improvement.

##### Increased Awareness About the Problem of Pain in Dementia

A majority of participants expressed positive views about the initiative, particularly about the manner in which the content was shared throughout the initiative and the resulting increased awareness about the problem of pain in dementia. One participant noted the importance of the information shared during the initiative:

Overall as a whole, I really liked it. I like the messaging that comes out of it, and I think it’s something really important to continue doing...I don’t think there’s a real understanding about pain and older adults. I like the messaging and I think it’s something that’s really important for us, as well as a society to promote.

Participants also expressed that the information shared during the initiative highlighted the experiences of older adults with dementia and pain. A person indicated that they thought the initiative was “great” because “the more information that can get out to caregivers, the better it will be for the residents [and] the people that have loved ones at home.” Furthermore, the initiative brought awareness to an underdiscussed problem of pain in dementia. For example, one participant stated:

Oh it’s good thing to put out here, something that people haven’t really thought about even the health care system and [pain in dementia] is a huge problem...so to bring it to the forefront is good, it needs to be brought to the forefront.

Overall, participant responses described a lack of prior understanding related to the experience of pain in dementia and the need for increased awareness about this topic.

Some individuals commented on the significance of the specific messages shared throughout the campaign in raising awareness about pain in dementia. For example, a participant noted the following:

I love what you’re doing. It’s a concern that everybody has, and why I call you a niche [because] it is a subject that people haven’t traditionally thought of.

Similarly, one participant expressed that the messaging on the videos were informational. This participant noted the following:

I guess one thing that stood out to me so much was the video of the man who seemed very angry. And so, I think back of all the people who’ve been labeled as the “angry person” could be the person in pain. And I think that message really hit me the most on pain.

Several participants expressed that the messaging shared during the initiative changed their perception about the problem of pain in people with dementia. A person commented on the videos:

You know one thing I did really like is the video with the gentleman that they thought was being kind of aggressive...I thought that video was really good but then on the other side [you] have the gentleman communicating how he is feeling. That was really good because that was really eye-opening.

##### Perceived Barriers to Pain Management

Knowledge users described various barriers that they have experienced in managing pain in their clinical practice or as a family member caring for a person living with dementia. The barriers encountered by knowledge users centered on inadequate pain assessments conducted in their practice or of their family member and relative living with dementia. For example, a health care professional reported the inadequate frequency of pain assessment in their facility:

You know, often in Ontario anyway, where I live, assessments in long-term care are done quarterly on clients and that’s, you know, that’ll be an all-encompassing assessment but certainly pain is part of that evaluation and, I mean, four times a year is not even close to being adequate to properly address and intervene if someone is living with pain.

In light of inadequate pain assessment practices, caregivers are often left to advocate for their family member or relative living with dementia. A caregiver in the study expressed the importance of families in advocating for assessment for their loved one:

I realized I don’t know if [my mother] might have tooth decay in her teeth [or if it] would be hurting her, and I don’t know how to figure that out. And I don’t think anyone else is checking. Yeah, because I think the lesson of my story here is that I am more likely to assess pain in my mom than her

care providers

Participants described lack of continuing education and awareness as significant barriers to improving practices in LTC settings. For instance, a participant indicated the following:

I think there’s certainly a lack of understanding and education by many health care professionals who are working with older adults who live with dementia, so I think a barrier is getting that education, well, I mean, it’s an opportunity for people to have more education but a barrier is kind of reaching the people.

Participants also indicated that the lack of education of the public at large was a significant barrier to improving current practices:

I think the barrier is the lack of education, the lack of knowledge that people with dementia can have pain and express it in a different way. So, I think that the lack of education of health care workers, but the public in general.

Insufficient education about the topic means that health professionals or caregivers of persons with dementia may not be aware of the signs and symptoms that can indicate pain. A caregiver in the study reflected on this notion and shared their lack of knowledge about the signs of pain in her mother living with dementia:

I mean I can think back to my mother who had two strokes. And after the second one, she used to scream all the time. She used to scream. And she never spoke to anyone, but she screamed. And when I questioned the doctor, if could she be in pain, he was so sure that she was not. It doesn’t manifest that way. And so, I think back like, “was she in pain all the time?” When she screamed louder when we came, was it a plea for help. I think lot about that.

##### Increased Knowledge and Changes in Behavior

Participants described the positive impact of the information on their knowledge and behavior. Some participants noted that the information shared during the initiative influenced their awareness and advocacy in their personal life or in clinical practice. Health professionals indicated that they found gaps in their own practice as a result of viewing the content shared in this initiative. A participant said the following:

It made me more aware of the gap in pain assessment with people like my mom. And then it also made me more aware that the onus then is on me as a caregiver to be doing it, and I feel woefully inadequate, that fact that I encountered the research has made me realize that it’s me doing the diagnostic [work] and I have no medical skill at all.

Greater insight into their own behavior was also reported by the participants. A clinician noted the following:

It also made me more aware for myself in my own practice...so that’s changed my practice in that way. To be more cognizant that behaviors the patient is displaying could be manifested due to pain. So, I need to ask the caregivers and ask the family, “Has he or she changed in behaviors in any way recently?” “Have you noticed, you know, a consistent type of behavior? Is it a specific time of day, is it related to something?” So that I could look more into pain and assessing it in a roundabout way, rather than asking them straight, “Do you think that your husband or wife is in pain?”

Other participants indicated that the initiative largely impacted their perspective on this issue (eg, “I would say it’s definitely changed the viewpoint”). Finally, participants indicated that they shared the knowledge they obtained with others:

I share things that you guys put out there, if you look back at my timeline, you’ll see periodically I make reference to you guys.

Similarly, one participant noted the following:

I forward all this information off to my colleagues that work in this [area] that are even more focused in the nursing aspect that would have a more direct impact on client care needs.

##### Value of Social Media as a Tool for Scientific Dissemination

Participants underscored the importance of leveraging social media to share research information in the age of the internet. Participants expressed that sharing evidence-based information fosters trust and credibility among users seeking information on social media platforms. For example, one participant noted the following:

I think it has a lot of positive benefits. Where to start? After this past year right, social media and technology were probably utilized more than they ever have been. So, I actually think it’s a really great way to disseminate that information. And then further to that too, because I've been following all your accounts for a while, just there again, yeah, the quality of the content is also really good. So, with that being said, it creates that credibility and that trust. And then that also really feeds into where social media sometimes isn’t the most trusted source. So, I do think that it’s been a really great way to get that information out there.

Participants also acknowledged the salience of social media platforms that needs to be further leveraged for scientific dissemination (eg, “social media certainly in light of COVID, like it is the way that information gets disseminated right now so I think it’s very appropriate and it is a strength that you’re on various platforms sharing resources”). Other participants expressed the importance of health initiatives in combating misinformation over the web:

I think a lot of times there’s a lot of misinformation for people and families and health care practitioners and [they] want to have some valid research-based information for best practices and when you do things like this and have it more accessible then it’s easier for people to gain the right information and make the best decisions for their family members or their clients that they can.

Other participants commented on the reach that social media platforms hold in raising awareness of issues in a short amount of time and connecting knowledge users across the world. One participant indicated the following:

It’s brilliant, social media has always been very, very good at distributing information. Creating awareness. Highlighting anything in social media catches the eye of the person. Because people are looking for answers.

In particular, one participant commented on the utility of Facebook targeting specific demographic groups:

I think, using social media is great, you know, the thing about Facebook, is that it is now an older person’s social media and people often think that that young people are caregivers of people with dementia. But it’s not always so–it’s old people who are caring for people with dementia. Facebook is good because most of us use Facebook.

##### Suggestions for Improvement

Finally, participants made suggestions about scaling and improving the initiative. One participant said the following:

I think it would be effective for you to reach out to the Alzheimer’s Society...I would love to see you partner with them in an active way on their media.

One participant suggested focusing on reaching individuals who may not be directly reached by the initiative. For example, one participant noted individuals who may not be using or cannot access social media platforms:

I think smaller communities don’t have the access or don’t know about it. I mean now we do have the Internet and that sort of thing which is fabulous but lots of time we don’t even know it’s out there.

As another example, one participant stated the following:

It’s the reachability...say like you know, even for me, it was from [someone else] that I found out about this, I have never seen it while using any of the social media account before. And like that largely depends on my browsing habits too right? But it’s the reachability that you guys have to concentrate on.

Other participants suggested incorporating information to traditionally delivered pain education:

And so, if there was a way, we could get this added into a pain curriculum, I think that would be excellent, because I never had it in my course, and I’ve learned from this.

In clinical practice, one participant expressed the following:

Well, I think, for so many people with dementia, their care is being provided by people with minimal education or I should say varying levels. If [people could get] a little certificate put into their little portfolios that would show, they have completed a course in pain assessment for people with dementia and that would somehow be an incentive for them as workers.

#### Social Media Comments

##### Overview

A total of 895 comments were included in the analysis. Most of the comments were retrieved from Facebook (eg, 822/895, 91.8%), followed by Twitter (eg, 68/895, 7.6%), and a small subset was comments by users on Instagram (eg, 5/895, 0.6%). A randomly selected 20% (179/895) subset was coded by a second coder to assess for agreement. The overall intercoder agreement was excellent (ie, *k*=0.80). Six themes emerged from the analysis: (1) positive comments in response to the initiative, (2) sharing their personal experiences in response to the content, (3) criticisms about and suggestions to improve pain management practices, (4) responses related to the COVID-19 pandemic, (5) negative comments in response to the content, and (6) advertisements.

##### Positive Comments in Response to the Initiative

Many users provided positive comments in response to the content. Facebook users expressed empathy in response to the content shared in the form of comments (eg, “I am praying for you” and “Amen”) and with the use of “emojis” (eg, praying emoji and red hearts). Other respondents commented on the importance of the initiative. For instance, a user on Facebook provided a comment noting the following:

The work you are doing is so important to all.

As another example, other users commended the initiative (eg, “such an important campaign #seepainmoreclearly”). Blog posts written by care partners in which they relayed their experiences as caregivers stimulated various positive discussions about the initiative. For example, a user commented “thanks for bravely sharing your experience with having a spouse with dementia, what a difficult journey, I enjoy [your books] so much!” in response to Louise Penny’s blog post shared on social media. As another example, a user expressed the following:

Thank you for this information which is truly important. Your story is important to open our minds to an area of Alzheimer’s that many did not think of.

##### Sharing Their Personal Experiences in Response to the Content

Other users responded to the initiative by sharing their personal experiences with the posted content. For example, one user stated the following:

My family went through this when my dad was only in his early 50s...no one had heard of Alzheimer’s...this disease is so sad and robs the family of so much. I’m glad there’s more support for families now, and wish we’d had more support when we needed it.

Another user expressed their experience of caring for their mother:

I wonder this all the time. My mom is nearly 101 and has dementia. She rarely expressed having pain. But how can she not when she is quite hunched over and has arthritis.

Other individuals provided additional commentary on the shared information. A user emphasized the importance of pain assessment:

People with dementia, people unable to clearly explain [their] pain, we need to look for clues daily that would help us determine if something out of the norm is going on.

##### Criticisms About and Suggestions to Improve Pain Management Practices

Critical comments about current practices or advocating for improved practices were also present. For instance, a user expressed the following in response to a post:

There is a problem with doctors overlooking any kind of pain.

Another user pointed out the issue of resource limitations in LTC facilities (eg, “staffing is a huge issue which needs a timely resolution”). Moreover, a user indicated that “seniors deserve much more than the less of minimum care they get.” Other users provided suggestions to improve practices:

There are so many kinds of dementia, each with their own stages. We need more access to good education and support for home care to help families cope.

Another user stated the following:

Pain assessment only on admission and then every 3 months? Pain must be assessed whenever there is the slightest indication of pain. A formal assessment every 3 months should reflect how effective the measures were.

##### Responses Related to the COVID-19 Pandemic

As the campaign occurred during the COVID-19 pandemic and disseminated vaccination information related to residents in LTC, a subset of the comments was related to the pandemic. Some users noted the negative ramifications of isolation in LTC (eg, “I am sick of the lockdowns in care homes. Not being able to take them out for a day for visits...is absolutely insane. They are suffering terribly over this, and it has to stop.”) Other comments denounced the significance of vaccines (eg, “the fake pandemic was created for the COVID vaccines”).

##### Negative Comments in Response to the Content

A subset of the comments expressed negative responses to the information shared. A few of these comments perpetuated stereotypes about older adults (eg, “people with dementia still feel pain?”) or dementia (eg, “reading this is enough to get dementia”). A user expressed disagreement with one of the information shared:

We assess residents for pain every time we see them. We are not stupid; we can tell when someone is in pain.

Do any of you work in LTC facility? Staff in dementia units are trained to look for any behavioral issues that may arise from pain.

##### Advertisements

Finally, a small subset of comments was from users advertising a product or information (eg, “Dementia and Alzheimer’s affects so many. Please don’t forget to check out my podcast, if you haven’t already” and “Can we help your loved one/care home/hospital/nursing home in UK with our free mp3s preloaded with the music of their own choice?? Contact me asap please click on the link.”).

### Responses to the Evaluation Questionnaires

The demographic characteristics of the knowledge users who responded to the questionnaires are presented in Multimedia Appendix 4. The majority of the survey respondents were caregivers of people living with dementia. Moreover, most respondents indicated that they had found the #SeePainMoreClearly campaign on Facebook and were living in Canada. Not all participants who responded to the survey completed all the questionnaires; therefore, the number of respondents is indicated in Tables 2 and 3. Table 2 outlines the respondents’ impression of the campaign. Across respondent groups, respondents endorsed a favorable impression of the campaign, a great likelihood of sharing information with others, and the use of social media for KM. Of note, the question regarding the new information provided by the content was rated lowest in each respondent group. Table 3 outlines the descriptive statistics based on the responses to the 6 general questions assessed by the IAM4all questionnaire. As noted in Table 3, responses indicated that most individuals (eg, 138/178, 77.5%) indicated the likelihood of using the information for themselves or someone else. Moreover, 77.1% (131/170) of the individuals expressed benefitting from the information shared during the initiative.

**Table 2:** Respondents’ impression of the campaign.

		Caregivers	Public	Health professional	Person living with dementia	Researcher	Policy maker
**Overall impression of the campaign^a^**							
	Value, mean (SD)	4.67 (1.63)	4.33 (1.49)	5.16 (1.72)	4.18 (1.72)	—^b^	—
	Value, n^c^	78	24	25	11	—	—
**Level of new information provided^d^**							
	Value, mean (SD)	3.91 (1.83)	4.21 (1.74)	4.04 (2.09)	3.50 (2.07)	—	—
	Value, n^c^	79	24	25	10	—	—
**Impact on information-seeking behavior^e^**							
	Value, mean (SD)	4.90 (1.89)	4.33 (1.76)	4.84 (1.82)	5.10 (2.33)	—	—
	Value, n^c^	79	24	25	10	—	—
**Using social media for scientific dissemination^f^**							
	Value, mean (SD)	5.48 (1.74)	5.00 (1.67)	5.44 (1.53)	5.00 (1.83)	—	—
	Value, n^c^	87	25	25	10	—	—

^a^Ratings are based on a 7-point scale, ranging from 1 (not at all) to 7 (very much): “Overall, how much did you like the image/clip/video?”

^b^Cell sizes <5 were not reported to protect the confidentiality of the participants.

^c^n=not all participants who responded to the survey completed all the questionnaires in the study; this number represents the number of participants who answered this question.

^d^“Did the video/image/clip provide you with new information?”

^e^“Are you likely to seek additional information about pain in dementia (and its assessment) as a result of watching this video/viewing this content?”

^f^“It takes an average of 17 years until professionals start to use important research results into their practice Do you think social media (e.g., Twitter) is a good way to spread important health information to caregivers of people with dementia/to people with dementia?”

**Table 3:** Survey responses to the IAM4all^a^ questionnaire (N=190).

Question			Respondents, n (%)
“**Why did you look for this information?”^b^**			
	To answer a question about the health of someone else (total number of respondents=190)	116 (61.1)	
	To satisfy my curiosity about a health matter (total number of respondents=172)	108 (62.8)	
	To find choices different from those given by a health professional (total number of respondents=172)	61 (35.5)	
	To follow-up on the information given by a health professional (total number of respondents=168)	52 (31)	
	To prepare myself before talking to a health professional (total number of respondents=168)	50 (29.8)	
	To help me decide if I should see a health professional (total number of respondents=169)	40 (23.7)	
	To answer a question about my health (total number of respondents=177)	30 (17)	
“**Did you find the information you were looking for?” (total number of respondents=181)**			
	Yes	73 (40.3)	
	Yes, but I did not understand it	15 (8.3)	
	No, I did not find it	72 (39.8)	
	No, but I found something else	21 (11.6)	
“**What did you think about this information?”^b^**			
	Now I want to learn more about this health matter (total number of respondents=161)	112 (69.6)	
	Now I know something new (total number of respondents=163)	79 (48.5)	
	I am reminded of something I already knew (total number of respondents=157)	76 (48.4)	
	This information says I did, or I am doing the right thing (total number of respondents=158)	66 (41.8)	
	Now I am reassured (total number of respondents=158)	66 (41.8)	
	I am not satisfied with this information (total number of respondents=153)	33 (21.6)	
	I think there is a problem with this information (total number of respondents=151)	14 (9.3)	
	I think this information could be harmful (total number of respondents=148)	2 (1.4)	
“**Did you or will you use the information for yourself” (total number of respondents=178)**			
	Yes	55 (30.9)	
	No, not for myself, but I used it for someone else	83 (46.6)	
	No, I did not use this information for myself or for someone else	40 (22.5)	
“**Did you (do you expect to) benefit from this information?” (total number of respondents=170)**			
	Yes	131 (77.1)	
	No	39 (22.9)	
**Did something negative come out from using this information? (total number of respondents=148)**			
	Yes	4 (2.7)	
	No	144 (97.3)	

^a^IAM4all: Information Assessment Method for all.

^b^Refers to participants who answered “Yes” with possible choices of yes, no, or possibly.

### Comparison of Web-Based Discussions About Pain in Dementia

The extent of discussions about pain in dementia was assessed by examining the total number of posts on Twitter and Facebook. After excluding posts pertaining to pain as a metaphor, unrelated to the problem of pain in dementia, shared by our own research group, and not in the English language, the number of tweets that were retained doubled from the precampaign period compared with during the campaign period (see Multimedia Appendix 5). A similar increase was also observed on Facebook. The number of discussions about pain in dementia decreased in the postcampaign period compared with during the campaign period (see Multimedia Appendix 5). The number of excluded posts in each period is summarized in Multimedia Appendix 6.

## Discussion

### Principal Findings

Social media allows the immediate dissemination of information to a large number of knowledge users. Although our relatively short pilot KM campaign (phase I) was successful, its longer-term continuation by researchers alone was not feasible owing to competing demands. As such, we partnered with a digital media company to launch phase II of the #SeePainMoreClearly campaign with expanded social media platform coverage. The goal of the initiative was to increase awareness of the challenges in the assessment and management of pain among people with dementia. We codeveloped key messages with partners (eg, health professionals, caregivers, and researchers) that served as the basis of our messaging.

Our findings demonstrated the effectiveness of social media KM methods in reaching very broad audiences over a 12-month period. Evidence of positive impact on knowledge users’ knowledge was demonstrated. The participants described positive perceptions in response to the information shared throughout the initiative. Survey respondents (eg, caregivers, health professionals, researchers, the public, and people with dementia) endorsed a favorable impression of the campaign. Overall, our initiative highlighted the advantages of using a science-media partnership (eg, collaboration with digital media experts, resources to develop tailored content and resources, and consistent dissemination of information). However, broad social campaigns require extensive resources and time commitments. We outline the recommendations to address these areas.

The current (phase II) initiative involving a science-media partnership was different in several regards from our pilot campaign that focused only on Twitter and a YouTube video. In comparison with the pilot campaign [9], visits to our web-based repository substantially increased in phase II, even when considering the difference in the lengths of the 2 campaigns. Phase II used extended evaluation metrics through the inclusion of interviews, in addition to social media analytics, questionnaires, and the analysis of social media responses over a 12-month period. Hashtag analytics on Twitter across the 2 phases were comparable, despite differences in the length of evaluation (eg, 5 months vs 12 months). We observed lower outside organization engagement during phase II of our initiative, which could have contributed to lower phase II engagement when accounting for the length of evaluation. Moreover, our social media presence was greater in phase II compared with our pilot campaign.

Overall, our findings demonstrated the effectiveness of social media KM methods in reaching very broad audiences quickly. In this study, we demonstrated success in directing knowledge users to a resource website with practical information for health professionals, caregivers, and people living with dementia. For instance, over 60,000 users from 82 countries viewed the web-based repository website over the 12-month period. In particular, the blog posts shared during the project attracted many readers. The blog web pages were viewed 59,919 times, providing evidence for their reach. This is comparable with other KM efforts showing the use of blogs as an engaging way of connecting to targeted users [32].

The reach and engagement of users on social media platforms also provided evidence for the success of the initiative in raising awareness about the problem of pain in dementia. Information shared on Facebook was successful at reaching a wide range of audience, with 1,313,485 people reached by the content shared on the Facebook page. However, it should be noted that some analytics (eg, reach and engagement) could not be obtained from social media sites (eg, LinkedIn) because these analytics were not provided by the platform. This limits our ability to deduce the full extent of engagement on these platforms. Nonetheless, the number of impressions on Facebook was the highest (eg, 4,100,000) compared with Twitter, Instagram, and LinkedIn. This is consistent with previous research showing extensive reach and engagement on Facebook compared with other platforms [6,33]. In contrast, Neil-Sztramko et al [34] conducted an awareness campaign targeted toward working caregivers and found that although Facebook posts generated the most reach, the quality of the engagement was low. The topic area, targeted audience, and length of evaluation could explain the differences between our findings and those of previous research. We observed the lowest reach and engagement on Instagram and LinkedIn. This is not surprising given that our primary audience comprised caregivers of people with dementia who tend to be older [35,36]. Older adults have shown a preference for Facebook in comparison with other social platforms [37]. Taken together, our findings suggest that Facebook may be the most suitable platform for disseminating information related to pain in dementia.

Our findings extend our pilot evaluation by including in-depth interviews with knowledge users who interacted with the campaign in addition to the analysis of social media analytics and evaluation questionnaires. Many interview participants indicated positive perceptions in response to the information shared throughout the initiative. Moreover, the participants expressed that the significance of the initiative was in bringing awareness to an underdiscussed problem. The interview participants acknowledged the utility of social media as a tool for scientific dissemination. Many participants noted the importance of leveraging social media to share research information. Other participants expressed the importance of health initiatives in combating misinformation over the web. Quantitative responses to the surveys also supported this view. Survey respondents endorsed the use of social media as a way of spreading important health information to caregivers of people with dementia and to people with dementia. We found evidence of the information and resources impacting users’ knowledge and behavior. For example, interview participants noted increased awareness and advocacy in their personal life and awareness of assessing for pain in their clinical practice. More importantly, participants frequently expressed intent to share the knowledge they obtained with others. This is consistent with the survey responses, indicating that a majority of respondents intended to use the information for themselves or others. Moreover, the respondents expressed benefitting from the information shared during the initiative.

The initiative stimulated web-based discussions about pain in dementia. Consistent with themes that emerged from social media responses on Twitter during the pilot campaign [9], many web users expressed positive comments in response to the initiative. In particular, expressions of support were prevalent in both the pilot and phase II of our campaign. Many web users who responded to the content disseminated during the campaign shared their personal experiences or added commentary to the posted content. In addition to sentiments of advocacy for better care, which was observed in our pilot campaign, social media comments in response to phase II of our campaign also highlighted criticism and suggestions to improve practices related to pain management in dementia. Users highlighted the need for access to continuing education and support for staff and families to increase the frequency of pain assessment in LTC. This demonstrated meaningful engagement by users in response to posts. Increased discussions also highlighted negative responses about the information shared (eg, perpetuating stereotypes about older adults and dementia), which was not observed in the pilot campaign. Differences in themes that emerged between the pilot and phase II could be due to the broader reach of phase II (eg, inclusion of Facebook), as the pilot campaign only examined responses using the #SeePainMoreClearly hashtag on Twitter. We also found increased discussion about pain in dementia during the initiative on Twitter and Facebook in comparison with before and after the initiative. However, this discussion was not sustained months following the end of the campaign. This highlights the importance of continuous and meaningful engagement to maintain gains made by an initiative. Given our methodology, we collected a substantially lower number of posts on Facebook in comparison with Twitter. This discrepancy is likely owing to the manual search that was conducted to obtain Facebook posts in comparison with the data that were easily pulled by Keyhole for Twitter posts. Depending on a user’s privacy settings, posts shared on personal or private accounts are excluded when conducting a general search on Facebook.

### Limitations and Directions for Future Research

A particular strength of this study is that the content shared during the initiative was developed collaboratively with family caregiver partners, researchers, media experts, and health care professionals. Various measures were also used to assess not only the web-based reach of the initiative but also the perceptions of knowledge users. Nonetheless, we acknowledge that the use of social media for KM research represents a new area that does not lend itself to strict scientific control in the evaluation of its effectiveness. We adapted a more nuanced approach in developing partnerships and using multimodal approaches to show the impact in different ways. Although we aimed to assess the impact on users’ knowledge and behavior, we did not directly evaluate behavior change. Future research should examine the associated behavior outcomes (eg, increased pain assessment) in addition to the spread of information (eg, analytics). It is also difficult to measure the impact of the initiative at an organizational or policy level. This could be an important avenue for future research. Individuals in our interview and surveys expressed their intention to use the information; however, the adoption and use of information was not obtained. Perhaps, a follow-up assessment of how or whether knowledge users applied learned information could be investigated. Moreover, the effectiveness of social and web modalities in creating meaningful changes in knowledge and behavior is subject to further examination. Notably, the landscape of social media is constantly changing, and researchers will need to adapt to these changes if they want to leverage this modality. For example, social media can be used to spread health misinformation [38]. Using social media for KM health efforts can also be used to combat false and unscientific web-based information. As such, ensuring the quality of scientific evidence disseminated on the web should be considered in the development of social media–based dissemination efforts. Although these findings may not be generalizable to other KM initiatives, our investigation adds to the growing body of knowledge leveraging social media as a KM tool.

### Recommendations

Our study highlighted the successes and challenges of social media KM initiatives. When comparing the pilot with the scaled initiative (phase II), there appears to be no considerable difference with regard to views, impressions, or reach when accounting for the length of time of each initiative (5 months vs 12 months). Although this scaled initiative demonstrated success in garnering a large reach across social media platforms, broad initiatives such as phase II of our campaign require extensive resources and time commitment, which may not be feasible for researchers in the long term. The following recommendations are proposed to assist researchers and partners who may be interested in developing and maintaining a web-based KM initiative:

1. Researchers could collaborate with established communication and marketing departments at their institution or other digital media partners to aid in digital and social media outreach of research information and internal and external advertising. Granting agencies may be able to allocate funds for such purposes.
2. Facebook and Twitter appear to be the most relevant platforms for KM for the topic of pain and dementia. However, researchers from other disciplines may find other platforms more helpful in reaching their target audience. For example, if a researcher’s area of research is adolescent mental health, Instagram or TikTok and the use of stories may increase engagement among knowledge users. Although we found success in using multiple social media platforms, it may be more sustainable to pool resources on 1 or 2 of the most used platforms based on the target audience.
3. Investments in targeted and paid advertisements can be an effective mechanism to increase the exposure and engagement of campaign posts at a low cost per engagement [39,40].
4. Our initiative highlighted the importance of codeveloping the initiative with partners of our intended target groups. Many of our partners developed blog posts about their lived experience, which generated the most engagement. As such, personalized blog posts and opportunities for knowledge users to share their experiences (ie, turning comments on) can be an easy and cost-effective way to stimulate discussions.
5. The success of our pilot initiative was largely influenced by partnering with professional organizations that helped disseminate information and resources to their audiences. Engagement from external organizations was lower during phase II of our initiative, potentially contributing to the overall decrease in engagement. We observed that external organizations were more inclined to retweet our content when it originated from our individual researcher accounts rather than from a generic SeePainMoreClearly social media account. Researchers should collaborate with organizations within their specific area of interest who can support in meaningfully engaging with their targeted audiences. Connecting with trusted messengers (eg, influencers, people with lived experience, and large followings) could help to penetrate targeted groups.

### Conclusions

We partnered with a digital media partner to launch phase II of the #SeePainMoreClearly campaign with expanded social media platform coverage. The goal of the initiative was to increase awareness of and provide resources related to the challenges in the assessment and management of pain among people with dementia. Although this scaled initiative demonstrated success in garnering large reach across social media platforms, broad initiatives such as phase II of our campaign (reported in this manuscript) require extensive resources and time commitments, which may not be feasible for researchers in the long term. Researchers should leverage collaborations with their institutions to aid in the digital media outreach of research information. Furthermore, granting agencies should consider allocating more funds for such KM purposes. Our initiative highlighted the importance of codeveloping KM efforts with the partners of our intended target groups and working with professional organizations to disseminate information to our target audience. Collaborations with people with lived experiences and professional organizations will be key to the success of any future KM effort. Our study adds to the growing body of knowledge that leverages social media as a KM tool.

## Appendix

Multimedia Appendix 1 Key points (cross-cutting messages).

Multimedia Appendix 2 Interview moderator guide.

Multimedia Appendix 3 Summary of the social media analytics for the #SeePainMoreClearly campaign.

Multimedia Appendix 4 Demographic characteristics of the survey respondents.

Multimedia Appendix 5 Number of posts on Twitter and Facebook about pain in dementia.

Multimedia Appendix 6 Number of excluded posts for each criterion.

## Abbreviations

IAM4all: Information Assessment Method for all
KM: knowledge mobilization
LTC: long-term care
