# Supplementary material for: The #SeePainMoreClearly Phase II Pain in Dementia Social Media Campaign: Implementation and Evaluation Study
Source: JMIR Aging. 2024 Feb 8;7:e53025. doi: 10.2196/53025 (PMC10884893; doi:10.2196/53025)
Supplement: Multimedia Appendix 1 [file aging_v7i1e53025_app1.docx]

## Multimedia Appendix 1

Key points (cross-cutting messages)

1. Pain is very common in long-term care (LTC) facilities. However, it is often underassessed, undermanaged, and undertreated largely because residents with moderate to severe are often unable to communicate the subjective state of pain and the intensity of that pain. (ALL)
2. There are validated observational tools for assessing pain in older adults with dementia. These tools rely on systematic observation of pain behaviors. (ALL)
3. Resource limitations in LTC are barriers to implementation of effective pain assessment and management (directed more to policy makers and administrators)
4. The PACSLAC scales are among the best validated tools for assessing pain in severe dementia (directed more for Health Care Professional [HCP])
5. Regular use of pain checklist tools, such as the PACSLAC scales, can help monitor pain levels and improve pain management and quality of life for older adults with dementia (HCP)
6. Health professionals sometimes misattribute behaviours, caused by pain, to psychiatric causes and try to manage them with dangerous psychiatric medication rather than pain medications. (HCP)
7. Treating pain appropriately for older adults with dementia can lead to improvements in their quality of life (ALL)
8. Pain in people with dementia should be assessed at least once a week and more often when pain is suspected (directed more to policy makers and administrators)
