# Supplementary material for: The #SeePainMoreClearly Phase II Pain in Dementia Social Media Campaign: Implementation and Evaluation Study
Source: JMIR Aging. 2024 Feb 8;7:e53025. doi: 10.2196/53025 (PMC10884893; doi:10.2196/53025)
Supplement: Multimedia Appendix 2 [file aging_v7i1e53025_app2.docx]

## Multimedia Appendix 2

**Interview Moderator Guide**

**General questions**

1. Why are you interested in the work that we have been doing with social media on pain in dementia (#SeePainMoreClearly)?
2. Do you know people with dementia?
   1. What is your relationship with the person with dementia?
3. What were some of your experiences in dealing with pain in individuals with dementia?
   1. How did you manage the pain experienced by your loved one?
   2. How did you assess and/or manage pain in your practice?
4. What are some barriers or challenges that you’ve experienced to improving pain assessment and management in your home/practice?
5. What are some of the strengths and weaknesses of this method (i.e., using social media to disseminate scientific information/resources)?
   1. As a caregiver/family member, what could be improved or what would you like to see being shared more from the initiative?
   2. As a health professional, what could be improved or what would you like to see being shared more from the initiative?
   3. As a policy maker, what could be improved or what would you like to see being shared more from the initiative?
   4. As an individual with dementia, what could be improved or what would you like to see being shared more from the initiative?
   5. As part of the general public, what could be improved or what would you like to see being shared more from the initiative?

**Quality of information/messages disseminated**

1. What specific messages did you see shared through the initiative?
   1. How often did you see it?
   2. What did you think of the messages/content?
2. What messages did you like best and what did you not like?
3. What are your overall thoughts on the information/resources being shared through the initiative?

**Impact on knowledge and behaviour**

**Public:**

1. In what ways have you used/implemented the information/resources provided through the initiative in your life and/or practice?
2. How has the information/resources shared changed your understanding of the way pain may affect people with dementia?

**Policy makers:**

1. How has the information/resources shared through the initiative influenced your understanding of the policy changes that are needed to help ensure that pain is assessed adequately in long term care (LTC) facilities?

**Health professionals:**

1. Has there been changes in your clinical practice or have you sought additional continuing education as a result of the information shared by the campaign?
2. How has the information/resources shared changed how you assess or identify pain in older adults with dementia?

**Family members/caregivers/person with dementia:**

1. In what ways has the initiative influenced your advocacy for improved pain assessment and management for yourself/your loved ones?
2. Have you discussed the information shared through our initiative with health professionals?
3. Has the information/shared through the initiative encouraged you to talk to a health professional about the possibility that some responsive behaviours may be due to pain
