# Supplementary material for: The #SeePainMoreClearly Phase II Pain in Dementia Social Media Campaign: Implementation and Evaluation Study
Source: JMIR Aging. 2024 Feb 8;7:e53025. doi: 10.2196/53025 (PMC10884893; doi:10.2196/53025)
Supplement: Multimedia Appendix 3 [file aging_v7i1e53025_app3.docx]

## Multimedia Appendix 3

|  |
| --- |
|  |
|  |

**Summary of the social media analytics for the #SeePainMoreClearly campaign.**

Time Period 1 = October 1, 2020-September 30, 2021. Time Period 2 = October 1,2020-November 2, 2020. Time Period 3 = November 3,2020-September 30, 2021.

“Facebook overall analytics” = generated from paid and unpaid impressions on Facebook and third-party websites such as newspaper websites that are part of the Facebook advertising display network across Canada.

“Twitter/Instagram/LinkedIn only analytics” = analytics based on unpaid posts circulated only on Twitter, Instagram, or LinkedIn.

“Twitter/LinkedIn ad analytics” = analytics based on paid posts circulated on Twitter and LinkedIn.

“Instagram ad analytics” = the analytics are based on paid posts that were posted on Facebook but reached Instagram audiences based on specified target audiences.

“Facebook only analytics” = based on paid and unpaid posts that were circulated only on Facebook.

CloudCampaign (i.e., third-party social media monitoring software) = these analytics are based on unpaid posts circulated individually on Twitter, LinkedIn, and Instagram. The Cloud Campaign analytics from Facebook includes analytics of paid and unpaid posts circulated only within Facebook.

On Instagram, the Time period 1 impressions only includes impressions for unpaid posts posted on Instagram. It does not include paid posts that were posted on Facebook but reached Instagram audiences.
