# Supplementary material for: The #SeePainMoreClearly Phase II Pain in Dementia Social Media Campaign: Implementation and Evaluation Study
Source: JMIR Aging. 2024 Feb 8;7:e53025. doi: 10.2196/53025 (PMC10884893; doi:10.2196/53025)
Supplement: Multimedia Appendix 4 [file aging_v7i1e53025_app4.docx]

## Multimedia Appendix 4

Demographic characteristics of survey respondents.

| Demographic |  | n (%) |
| --- | --- | --- |
|  |  |  |
| **Group (n**^c^ **= 675)** | | |
|  | Informal/unpaid caregiver of PwD^a^ | 447 (66.20) |
|  | Public | 101 (15.00) |
|  | Health professional | 63 (9.30) |
|  | Person with dementia | 53 (7.90) |
|  | Researcher working in the field of dementia and cognitive decline | 7 (1.00) |
|  | Policymaker | 4 (0.60) |
| **Age M (SD; n = 506)** |  | 67.39 (11.83) |
| **Gender (n = 534)** |  |  |
|  | Female | 476 (89.13) |
|  | Male | 54 (10.11) |
|  | Other | 4 (0.75) |
| **Mode of Locating the SPMC^b^ Campaign (n = 608)** |  |  |
|  | Facebook | 523 (86.00) |
|  | Twitter | 33 (5.40) |
|  | Family/friend | 16 (2.60) |
|  | SPMC website | 14 (2.30) |
|  | Other | 12 (2.00) |
|  | Instagram | 9 (1.50) |
|  | YouTube | 1 (0.20) |
| **Country (n = 590)** |  |  |
|  | Canada | 549 (93.05) |
|  | United States of America | 24 (4.07) |
|  | United Kingdom | 6 (1.02) |
|  | Other | 11 (1.85) |
| **^a^**PwD = Person living with dementia.  ^b^SPMC = See Pain More Clearly;  ^c^n = Not all participants who responded to the survey completed all the questionnaires in the study; this number represents the number of participants who answered this question. | | |
