# Supplementary material for: The #SeePainMoreClearly Phase II Pain in Dementia Social Media Campaign: Implementation and Evaluation Study
Source: JMIR Aging. 2024 Feb 8;7:e53025. doi: 10.2196/53025 (PMC10884893; doi:10.2196/53025)
Supplement: Multimedia Appendix 5 [file aging_v7i1e53025_app5.docx]

Multimedia Appendix 5

The figure illustrates the number of posts on Twitter and Facebook about pain in dementia (excluding posts by our team). Precampaign period=March 1, 2020, to September 30, 2020; during the campaign=October 1, 2020, to September 30, 2021; and postcampaign period=October 1, 2021, to April 30, 2022.
