# Supplementary material for: The #SeePainMoreClearly Phase II Pain in Dementia Social Media Campaign: Implementation and Evaluation Study
Source: JMIR Aging. 2024 Feb 8;7:e53025. doi: 10.2196/53025 (PMC10884893; doi:10.2196/53025)
Supplement: Multimedia Appendix 6 [file aging_v7i1e53025_app6.docx]

## Multimedia Appendix 6

Number of excluded posts for each criterion.

|  | Pertaining to pain as a metaphor | Unrelated to the problem of pain in dementia | Posts shared by our own research group | Not in the English language |
| --- | --- | --- | --- | --- |
| **Twitter** |  |  |  |  |
| Pre-campaign | 1,098 | 2,830 | 47 | 3 |
| During the campaign | 1,891 | 5,092 | 358 | 17 |
| Post campaign | 910 | 2,103 | 40 | 12 |
| **Facebook** |  |  |  |  |
| Pre-campaign | 5 | 20 | 1 | 0 |
| During the campaign | 18 | 77 | 44 | 0 |
| Post campaign | 13 | 55 | 4 | 0 |
